# Trends in hospital discharge outcomes among high-risk Medicare beneficiaries before and during the COVID-19 pandemic

**DOI:** 10.1093/haschl/qxaf056

**Published:** 2025-03-18

**Authors:** Anthony I Roberts, Christopher M Santostefano, Zihan Chen, Brian E McGarry, Elizabeth M White, Linda J Resnik, Fangli Geng, David C Grabowski, Momotazur Rahman

**Affiliations:** Department of Health Services, Policy, and Practice, Brown University School of Public Health, Providence, RI 02903, United States; Department of Health Services, Policy, and Practice, Brown University School of Public Health, Providence, RI 02903, United States; Department of Health Services, Policy, and Practice, Brown University School of Public Health, Providence, RI 02903, United States; Division of Geriatrics and Aging, Department of Medicine, University of Rochester, Rochester, NY 14642, United States; Department of Health Services, Policy, and Practice, Brown University School of Public Health, Providence, RI 02903, United States; Department of Health Services, Policy, and Practice, Brown University School of Public Health, Providence, RI 02903, United States; Department of Health Services, Policy, and Practice, Brown University School of Public Health, Providence, RI 02903, United States; Department of Health Care Policy, Harvard Medical School, Boston, MA 02115, United States; Department of Health Services, Policy, and Practice, Brown University School of Public Health, Providence, RI 02903, United States

**Keywords:** COVID-19, comorbidities, Medicare, Medicare Advantage, Traditional Medicare, hospital discharge outcomes, post-acute care, mortality, length of stay, home health care, skilled nursing facilities

## Abstract

**Introduction:**

Medicare beneficiaries face significant health risks and care disruptions during public health emergencies, but little is known about how care patterns evolved throughout the COVID-19 pandemic or differed between traditional Medicare (TM) and Medicare Advantage (MA).

**Methods:**

Using Medicare claims data for over 20 million hospital discharges during 2018-2022, we examined trends in hospital length of stay, discharge disposition, and mortality among beneficiaries with 5 major comorbidities (dementia, diabetes, congestive heart failure, hip fracture, and stroke), stratified by COVID status and payer type.

**Results:**

We found that COVID patients initially experienced substantially longer hospital stays (8.3 vs 4.6 days) and higher 30-day mortality (34% vs 5%) compared to patients without COVID. MA beneficiaries showed consistently higher home health utilization but similar mortality patterns to TM enrollees. By mid-2022, most outcome differences had converged between COVID and non-COVID patients, suggesting health system adaptation to the pandemic.

**Conclusion:**

Our findings highlight how the pandemic was associated with shifts toward home-based post-acute care, emphasizing the need for policies supporting home-based care infrastructure and flexible care delivery models that could help health systems better adapt during future public health emergencies.

## Introduction

The COVID-19 pandemic significantly disrupted healthcare delivery and outcomes for older adults, one of the most vulnerable populations to the virus.^1,2^ Prior studies have highlighted the pandemic's impact on hospital-based care,^3-5^ documenting increased mortality rates,^6,7^ and notable changes in discharge disposition patterns among the Medicare population.^4,8,9^ However, several important knowledge gaps remain in our understanding of how hospital care delivery and post-acute care transitions evolved from the height of the pandemic and as the pandemic waned.

Medicare beneficiaries, who account for approximately 19% of the U.S. population but represented nearly 80% of COVID-19 deaths during the initial waves of the pandemic, faced unique challenges during the pandemic.^10,11^ Early reports documented substantial disruptions in care access, with hospital admission volumes dropping by 32%-43% during March-April 2020 compared to pre-pandemic levels.^3,12^ These disruptions particularly affected older adults with chronic conditions requiring ongoing medical management, heightening concerns about care quality and continuity for this population.^13,14^

Post-acute care utilization patterns also shifted dramatically during the pandemic.^8^ A notable change was a shift away from post-acute care provided in skilled nursing facilities (SNFs), toward home-based care. Early in the pandemic, discharges to SNFs decreased (from roughly 19% of Medicare hospital discharges in 2019 to about 14% in late 2020), while discharges home with home health care rose, making home health the dominant post-acute care setting by mid-2020.^8,15^ These shifts were influenced by multiple factors, including infection concerns in congregate settings, workforce shortages, and changing patient and provider preferences. Although some rebound in SNF admissions for post-acute care has occurred, evidence suggests the pandemic accelerated a preexisting trend toward utilization of home-based care.^8^ Importantly, while discharge dispositions to SNFs began to rebound by 2022, it remains unclear whether this recovery was uniform across all patient populations and payer types, particularly for high-risk individuals with complex chronic conditions who often require more intensive post-acute care services.^15^

Even before the pandemic, Medicare post-acute care utilization was undergoing significant transformation due to payment policy changes. In October 2019, the Centers for Medicare and Medicaid Services (CMS) implemented the Patient-Driven Payment Model (PDPM) for SNFs, which shifted from therapy volume-based payments to a case-mix classification system based on patient characteristics.^16^ Similarly, in January 2020, CMS introduced the Patient-Driven Groupings Model (PDGM) for home health, replacing the previous episode-based payment approach with a patient needs-based system.^17^ These reforms created financial incentives that likely influenced discharge patterns. For instance, PDPM encouraged SNFs to admit more medically complex patients while shortening lengths of stay, while PDGM restructured home health payments based on patient characteristics rather than service volume.^18^ The timing of these payment reforms, implemented just before or during the early stages of the pandemic, creates an important backdrop for understanding the evolution of discharge disposition patterns during this period.

While prior research has explored overall COVID-19 effects on healthcare utilization and outcomes, less is known about how these trends differed between patients hospitalized with and without COVID or between traditional Medicare (TM) beneficiaries and Medicare Advantage (MA) enrollees. With over half of all Medicare beneficiaries being enrolled in MA,^19^ understanding differences in care patterns and outcomes between these payer types is increasingly important for Medicare policy development. Historical evidence indicates that MA enrollees typically receive less post-acute care overall, including fewer days in SNFs, lower use of inpatient rehabilitation facilities, and reduced home health care utilization, compared to TM beneficiaries, yet experience lower hospital readmission rates and higher rates of successful discharge to the community.^20,21^ Given these historical differences, MA plans, with their capitated payment structure, flexible benefit design and incentives to manage utilization, may have been able to respond differently than TM to pandemic-related challenges.^22^

Additionally, most existing studies have focused on the acute phase of the pandemic (2020-2021), with limited investigation into how trends evolved as healthcare systems adapted and the pandemic waned.^8^ Understanding these longer-term patterns is crucial for informing health system resilience and preparedness for future public health emergencies.

This study aims to address these knowledge gaps by examining trends in hospital length of stay, discharge disposition patterns, and mortality outcomes among Medicare beneficiaries with 5 common and high-risk conditions: dementia, diabetes, congestive heart failure (CHF), hip fracture, and stroke. Using Medicare claims data from 2018 to 2022, we examined how these outcomes differed by COVID-19 status and Medicare payer type, and how these patterns evolved throughout the pandemic period.

## Methods

### Study population and data

We analyzed unique hospital discharges identified using the Medicare Provider Analysis and Review (MedPAR) file, between January 2018 and December 2022. MedPAR aggregates inpatient claims to the stay-level for 100% of Medicare beneficiaries who had an inpatient admission to an acute care, rehabilitation, or psychiatric hospital. Claims were linked to the Master Beneficiary Summary File (MBSF) using unique patient identifiers to obtain demographics, MA enrollment in a given month, Medicaid eligibility, and the date of death. We classified beneficiaries as MA enrollees if they were enrolled in an MA plan during the month of their hospital discharge. The study included beneficiaries Medicare beneficiaries aged 65 and older who were discharged with at least one of the following diagnoses documented during their hospitalization: Alzheimer's disease or related dementias, diabetes, CHF, hip fracture, or stroke. Diagnoses were identified from all 25 diagnosis fields in the MedPAR record of the index hospitalization using the enhanced Chronic Conditions Data Warehouse International Classification of Diseases, Tenth Revision (ICD-10) code algorithms published in 2020.^23^ While all beneficiaries in the sample qualified for Medicare based on age, a small proportion (<2%) also qualified through end-stage renal disease entitlement. We excluded beneficiaries who had used inpatient services in the previous 6 months to focus on incident hospitalizations. COVID-19 status was determined using ICD-10 diagnosis codes documented in any of the 25 diagnosis fields in MedPAR during the index hospitalization. Following the approach used by CMS, we used codes U07.1 and B97.29 prior to the introduction of code U07.1 in April 2020, and U07.1 only from April 2020 onward.^24^ We defined the pandemic period as beginning in March 2020 during the first U.S. wave.

### Key measures

Our outcomes include hospital length of stay (days between the date of admission and discharge), discharge destination identified on the inpatient claim (home/self-care, skilled nursing facility, home health services, or inpatient rehabilitation facility), and 30-day all-cause mortality (assessed using MBSF death dates).

### Analytic approach

We estimated trends in monthly discharge dispositions using regression models to generate risk-adjusted probabilities of an outcome in each month, controlling for age, sex, race, dual eligibility status, Charlson Comorbidity Index computed from hospital diagnoses, the aforementioned comorbidity diagnoses, and hospital fixed effects. The models included interaction terms between month-year and payer type, and between month-year and COVID-19 status, allowing us to estimate differential trends by payer type and COVID-19 diagnosis. We used linear regression for hospital length of stay and logit models for binary outcomes.

### Limitations

Our results should be interpreted within the context of certain limitations. First, while we controlled for observable patient characteristics and hospital fixed effects, unobserved differences between MA and TM beneficiaries may influence our findings. Second, our analysis focused on beneficiaries with specific comorbidities, and results may not generalize to the broader Medicare population. Third, because our identification of comorbidities relies on Medicare claims data, it may be subject to coding differences between TM and MA. Given that MA plans have been shown to exhibit higher coding intensity, it is possible that some observed differences in comorbidity burden reflect differential coding rather than true clinical differences.^25,26^ Fourth, our study relies on MedPAR data, which likely undercounts MA beneficiary hospitalizations because hospitals don't directly bill CMS for these stays. While we used MBSF enrollment data to classify MA status, the underlying sample may not fully represent all MA hospitalizations. This reporting bias varies by hospital characteristics and service types, and the extent of underreporting remains uncertain, as noted in MedPAC analyses.^27^ Finally, our data cannot fully capture the impact of important factors like vaccination status or specific therapeutic interventions that emerged during the pandemic.

## Results

From January 2018 to December 2022, we observed 20 991 657 hospital discharges of Medicare beneficiaries with dementia, diabetes, CHF, hip fracture, or stroke, including 1 123 615 discharges for patients with COVID (Table 1). Patient characteristics and outcomes differed between the pre-pandemic and pandemic periods. Compared to all pre-pandemic patients, COVID patients were more likely to be dual-eligible (30% vs 23%) and have diabetes (64% vs 57%); and were less likely to have a stroke (4.6% vs 9.5%) or hip fracture (2.1% vs 6.4%), or be discharged home with self-care (34% vs 43%). COVID patients experienced longer hospital stays (mean, 8.7 vs 5.1 days), were more likely to require intensive care unit admission (40% vs 33%), and had nearly triple the 30-day mortality rate (27% vs 9.7%) than pre-pandemic patients. (Table 1). Total discharge volume dropped during the initial wave of the pandemic, reaching a low of 238 000 discharges in April 2020 compared to typical monthly discharges of 340 000-370 000 pre-pandemic (Figure S1). The share of discharged patients with the 5 diagnoses remained relatively stable from the pre-pandemic to pandemic periods, with diabetes being the most common diagnosis (56%-58%) (Figure S2).

**Table 1.** Characteristics of Medicare beneficiaries discharged from inpatient hospitals with diagnoses of dementia, diabetes, congestive heart failure, hip fracture, or stroke, 2018-2022.

	Pre-pandemic period	Pandemic period	
Characteristic	All patients *N* = 9 493 647	Patients without COVID *N* = 10 374 395	Patients with COVID *N* = 1 123 615
Patient characteristics			
Age, median (Q1, Q3)	78 (71, 85)	78 (72, 85)	78 (72, 85)
Female, %	54%	54%	50%
White, %	75%	75%	67%
Black, %	11%	11%	14%
Dual-eligible, %	23%	23%	30%
Medicare fee-for-service, %	64%	56%	53%
Hospitalization			
Intensive Care Unit Use, %	33%	34%	40%
Length of stay, mean (SD)	5.1 (5.5)	5.4 (6.4)	8.7 (9.7)
Comorbidity conditions			
Dementia, %	24%	23%	30%
Diabetes, %	57%	56%	64%
Congestive heart failure, %	41%	42%	34%
Hip fracture, %	6.4%	7.5%	2.1%
Stroke, %	9.5%	11%	4.6%
Multiple (≥2) selected comorbidities^a^	33%	34%	31%
Charlson comorbidity score, mean (SD)	3.01 (1.82)	3.06 (1.84)	2.93 (1.77)
Discharge locations			
Home/self-care, %	43%	44%	34%
Home health service, %	18%	21%	19%
Inpatient rehabilitation, %	4.4%	4.9%	2.3%
SNF/Swing bed, %	24%	20%	20%
Death within 30 days of discharge, %	9.7%	11%	27%

Unadjusted percentages and means (with standard deviations) for demographic, clinical, and discharge characteristics of Medicare beneficiaries aged 65 and older with at least 1 of 5 selected comorbidities. Data stratified by time period and COVID-19 status. COVID-19 status determined using ICD-10 diagnosis codes (U07.1 and B97.29 before April 2020; U07.1 only thereafter) documented in any of the 25 diagnosis fields in MedPAR. ^a^Multiple selected comorbidities refer to beneficiaries with 2 or more of the 5 study comorbidities (dementia, diabetes, congestive heart failure, hip fracture, or stroke).

**Figure 1.**
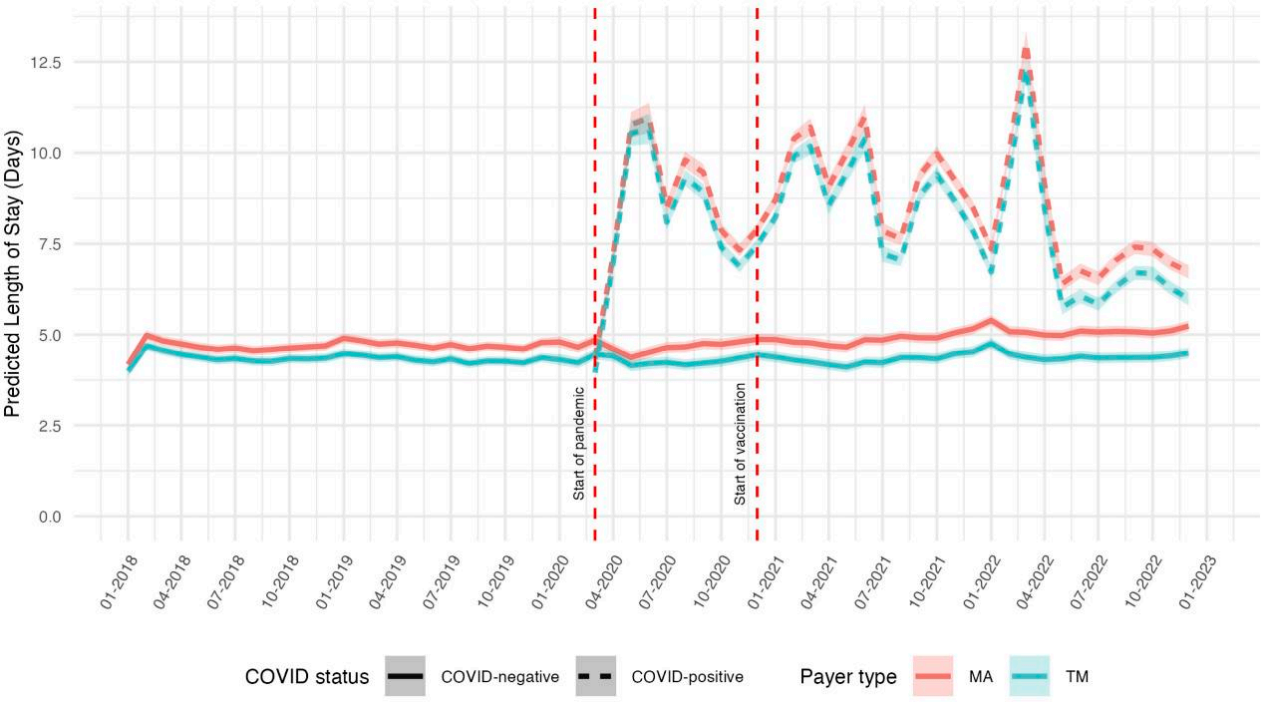
Risk-adjusted hospital length of stay among Medicare beneficiaries with high-risk comorbidities, by COVID-19 status and payer type, 2018-2022. Estimates of the monthly predicted average hospital length of stay in days are from linear regression models with hospital fixed effects, adjusted for age, sex, race, dual eligibility status, Charlson Comorbidity Index, and comorbidity diagnoses. Shaded areas represent 95% confidence intervals. First vertical dashed line indicates the onset of the COVID-19 pandemic in March 2020; second vertical dashed line indicates the introduction of COVID-19 vaccines in December 2020.

During the pandemic, COVID patients had longer length of stay (8.3 days, 95% CI: 8.1-9.5) compared to non-COVID patients (4.6 days, 95% CI: 4.5-4.8) (Figure 1). MA beneficiaries consistently had modestly longer lengths of stay than TM beneficiaries, both among COVID patients (8.6 days, 95% CI: 8.3-8.8 for MA vs 8.0 days, 95% CI: 7.8-8.2 for TM) and non-COVID patients (4.9 days, 95% CI: 4.7-5.0 for MA vs 4.3 days, 95% CI: 4.2-4.5 for TM). While the introduction of COVID-19 vaccines in late 2020 did not appear to alter the length of stay patterns, by mid-2022, length of stay for non-COVID patients returned near pre-pandemic levels, while the length of stay for COVID patients showed a gradual reduction but remained elevated compared to pre-pandemic levels.

**Figure 2.**
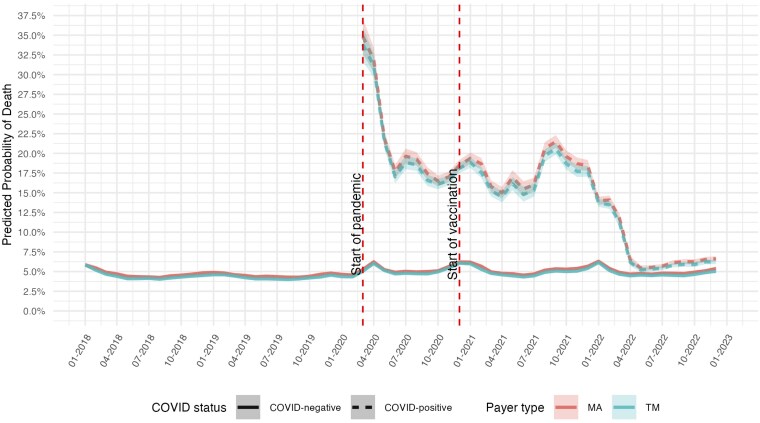
Risk-adjusted 30-day all-cause mortality rates among Medicare beneficiaries with high-risk comorbidities, by COVID-19 status and payer type, 2018-2022. Estimates of the monthly predicted probability of 30-day all-cause mortality are from logit models with hospital fixed effects, adjusted for age, sex, race, dual eligibility status, Charlson Comorbidity Index, and comorbidity diagnoses. Shaded areas represent 95% confidence intervals. First vertical dashed line indicates the onset of the COVID-19 pandemic in March 2020; second vertical dashed line indicates the introduction of COVID-19 vaccines in December 2020. Mortality assessed using death dates from the Medicare Master Beneficiary Summary File.

Figure 2 plots the trends in risk-adjusted 30-day mortality rates. 30-day mortality among patients without COVID remained roughly the same at around 5.0% throughout the entire study period. However, the mortality rate among COVID patients was over 34% (95% CI: 33-37) at the start of the pandemic. It declined to around 18% (95% CI: 17-19) by June 2020, fluctuated until the third quarter of 2021, and then declined steadily until May 2022, when it reached a new steady state. By the end of the study period, the mortality rates were 6.25% among COVID patients and 5% among non-COVID patients. We did not observe significant differences in mortality rates between TM and MA enrollees.

**Figure 3.**
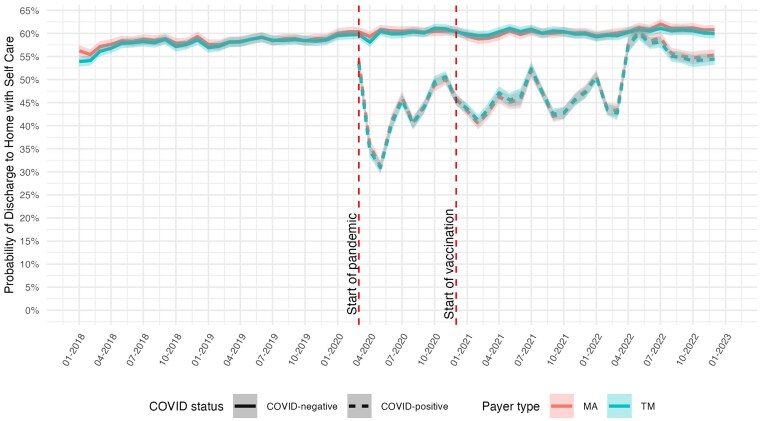
Risk-adjusted probability of discharge to home with self-care among Medicare beneficiaries with high-risk comorbidities, by COVID-19 status and payer type, 2018-2022. Estimates of the monthly predicted probability of discharge to home with self-care are from logit models with hospital fixed effects, adjusted for age, sex, race, dual eligibility status, Charlson Comorbidity Index, and comorbidity diagnoses. Shaded areas represent 95% confidence intervals. First vertical dashed line indicates the onset of the COVID-19 pandemic in March 2020; second vertical dashed line indicates the introduction of COVID-19 vaccines in December 2020.

Figure 3 shows the adjusted probability of discharge to home with self-care, which increased steadily throughout the entire study period for non-COVID patients from about 56% (95% CI: 55-57) in January 2018 to about 61% (95% CI: 60-62) in December 2022. For COVID patients, the proportion discharged to home fluctuated between 40% and 50% from the second quarter of 2020 to the first quarter of 2022, improving from early pandemic lows of 32%. The proportion of COVID patients discharged to home reached its highest level in May 2022 when it became similar to that of non-COVID patients and then declined slightly to 55% (95% CI: 54-56) by the end of the study period. MA and TM beneficiaries showed similar rates of discharge to home.

**Figure 4.**
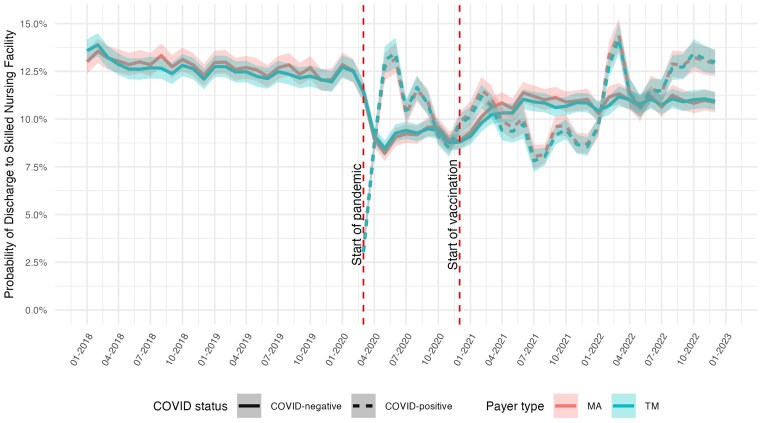
Risk-adjusted probability of discharge to skilled nursing facilities among Medicare beneficiaries with high-risk comorbidities, by COVID-19 status and payer type, 2018-2022. Estimates of the monthly predicted probability of discharge to skilled nursing facilities from logit models with hospital fixed effects, adjusted for age, sex, race, dual eligibility status, Charlson Comorbidity Index, and comorbidity diagnoses. Shaded areas represent 95% confidence intervals. First vertical dashed line indicates the onset of the COVID-19 pandemic in March 2020; second vertical dashed line indicates the introduction of COVID-19 vaccines in December 2020.

Figure 4 plots the proportion of patients discharged to SNFs. Before the start of the pandemic, the share of patients discharged to SNFs declined gradually and was about 12.5% in the first quarter of 2020. Consistent with prior studies,^16,28^ we did not observe any significant change with the implementation of PDPM in October 2019.. The proportion discharged to SNF among non-COVID patients decreased sharply to around 9% (95% CI: 8.7-9.6) in April 2020 and remained at that level until December 2020, when the COVID vaccines first became available. This rate increased steadily during the first 2 quarters of 2021 and then remained stable at around 11% during the remaining 6 quarters of the study. The proportion of COVID patients discharged to SNF was about 3% at the start of the pandemic, fluctuated between 7% and 14% during the pandemic months, and was 13% (95% CI: 12.4-13.6) in December 2022. The rates for MA and TM were fairly comparable and tracked closely to each other during the entire study period.

**Figure 5.**
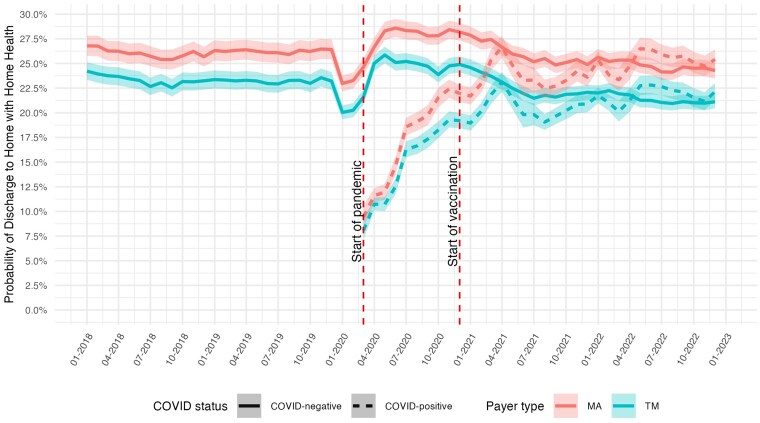
Risk-adjusted probability of discharge to home with home health services among Medicare beneficiaries with high-risk comorbidities, by COVID-19 status and payer type, 2018-2022. Estimates of the monthly predicted probability of discharge to home with home health services are from logit models with hospital fixed effects, adjusted for age, sex, race, dual eligibility status, Charlson Comorbidity Index, and comorbidity diagnoses. Shaded areas represent 95% confidence intervals. First vertical dashed line indicates the onset of the COVID-19 pandemic in March 2020; second vertical dashed line indicates the introduction of COVID-19 vaccines in December 2020.

Figure 5 illustrates the risk-adjusted likelihood of being discharged to home with home health services over the study period. For TM enrollees without COVID, the proportion discharged with home health care remained largely stable at 23% prior to the pandemic. However, this rate dropped to 20% (95% CI: 19.3-20.7) in January 2020, coinciding with the implementation of the PDGM. It then rose with the onset of the pandemic, peaking at 26% (95% CI: 25.0-26.7) in May 2020, before gradually declining over the pandemic's duration and stabilizing at 21% by the third quarter of 2021. Among TM enrollees with COVID, the proportion discharged with home health care started at approximately 8% (95% CI: 7.1-9.1) at the onset of the pandemic, increased steadily over time, and aligned with the rate for non-COVID patients by the fourth quarter of 2021. MA enrollees, regardless of COVID-19 diagnosis, were consistently more likely than TM enrollees to be discharged with home health care. However, MA enrollees exhibited similar trends to TM enrollees throughout the study period.

Rates of discharge to inpatient rehabilitation facilities remained low throughout the study period but were slightly more common among non-COVID patients than COVID patients during the pandemic. TM beneficiaries were more likely than MA enrollees to be admitted to these settings across the study period (Figure S3).

## Discussion

Our analysis of over 20 million hospital discharges highlights key differences in discharge dispositions and outcomes between hospitalized Medicare beneficiaries with and without COVID-19 before and during the pandemic. During the pandemic, COVID patients experienced longer hospital stays, lower likelihood of discharge to home with self-care, and higher mortality rates compared to patients without COVID. However, these patterns for the 2 groups generally converged by the second quarter of 2022. Additionally, we observed significant shifts in post-acute care utilization patterns, with a notable transition toward home health care that began to converge between COVID and non-COVID patients by early 2021, coinciding with the widespread availability of COVID-19 vaccines.

A key contribution of this study is the observation of differences in outcomes for MA enrollees and TM beneficiaries. While both MA and TM beneficiaries showed similar overall trends, MA enrollees, both those with and without COVID, on average experienced modestly longer hospital stays. Additionally, MA beneficiaries demonstrated higher utilization of home health services compared to TM beneficiaries, whereas TM enrollees had greater use of inpatient rehabilitation facilities. Interestingly, this finding contrasts with earlier studies that have found MA enrollees to have lower rates of home health care, attributed to more restrictive prior authorization processes and frequent recertification requirements for home health services.^29-32^ This contrast could be driven by our focus on hospitalizations with 5 selected comorbidities. The differences in discharge disposition patterns between MA and TM beneficiaries, even during a public health crisis, suggest that program-level incentives and care management approaches may influence service use.

Mortality rates among hospitalized COVID patients fluctuated over time, declining significantly after the first wave of the pandemic as knowledge and therapeutics for inpatient management of COVID-19 improved, and then again with the introduction of COVID-19 vaccines in December 2020.^33^ Mortality increased again during the Delta variant surge in the summer of 2021,^34^ and then declined again in late 2021 and early 2022 with the emergence of the Omicron variants which were more contagious but less lethal than prior strains.^35^ Other factors, including broader population immunity due both to vaccination and natural infection and improved clinical management of COVID-19, including the emergence of antivirals and monoclonal antibodies, contributed to this transition to the “new normal.”^36-39^ Importantly, the similar mortality patterns observed among MA and TM beneficiaries, despite some differences in post-acute care use, suggest that insurance type had little influence on mortality, underscoring the dominant clinical impact of COVID-19 on high-risk populations regardless of payer type.

Our study also identified a significant shift in post-acute care patterns. Comparing before and after the pandemic, we observed a decline in discharges to SNF, decreasing from 11.9% to 9.6% among non-COVID patients. Concurrently, the proportion of patients discharged to home—both with and without home health care—increased. These findings point to a broader trend away from SNFs during the pandemic, likely driven by concerns about infection risk and evolving care preferences.^9^

These trends in discharge patterns have several policy implications for Medicare. First, the shift toward home-based post-acute care highlights the need for appropriate infrastructure and reimbursement to support post-acute care in the home setting.^40,41^ The recent change in Medicare's home health payment model to the PDGM, which shifted reimbursement to be based on patient clinical characteristics rather than service volume, needs ongoing evaluation to ensure it incentivizes appropriate home-based care for high-risk patients.^42^ Second, access to telehealth and remote monitoring services could potentially help support safe transitions to home for certain patients with chronic illness. During the pandemic, CMS temporarily expanded telehealth coverage, allowing providers to virtually engage patients at home when in-person visits posed risks​.^43^ Making some of these telehealth flexibilities permanent, with appropriate safeguards and ongoing evaluation of their effectiveness, may help improve continuity of care for recently discharged patients by enabling timely virtual check-ins, monitoring of symptoms, and follow-up consultations​. Third, investment in the home-based care workforce is critical. The pandemic exacerbated nationwide shortages of nurses, therapists, home health aides, and other direct care workers, straining the capacity of home health providers.^44^ Policymakers may need to bolster workforce development programs and incentives to attract and retain qualified home health professionals. For example, several states have used American Rescue Plan Act funds and Medicaid waivers to strengthen their direct care workforce, and similar investment strategies could be considered within Medicare (or through cross-payer initiatives) to ensure an adequate supply of home-based care services for Medicare beneficiaries.^45^

Our findings also highlight the importance of having comprehensive discharge and transitional care plans in place for both TM and MA populations, particularly those with chronic illness after hospitalization for COVID-19. Value-based care initiatives like the Hospital Readmissions Reduction Program incentivize improved discharge planning and care coordination for conditions common in our study cohort.^46^ Policy efforts could focus on strengthening evidence-based discharge planning and post-discharge follow-up across both payment models, perhaps by enhancing reimbursement for transitional care management services. Ultimately, our findings suggest that quality of care transitions, rather than payer type, are associated with outcomes for vulnerable patient populations during public health emergencies.

In conclusion, our analysis highlights the evolving impact of the COVID-19 pandemic on hospital discharge dispositions and post-acute care patterns among Medicare beneficiaries. While differences between COVID and non-COVID patients attenuated over time, the pandemic accelerated a shift away from post-acute care provided in SNFs toward home-based care. These findings underscore the need for continued monitoring and adaptive health policies to support vulnerable populations in the face of future public health challenges.

## Supplementary Material

qxaf056_Supplementary_Data
