# Supplementary material for: Trends in hospital discharge outcomes among high-risk Medicare beneficiaries before and during the COVID-19 pandemic
Source: Health Aff Sch. 2025 Mar 18;3(4):qxaf056. doi: 10.1093/haschl/qxaf056 (PMC11970246; doi:10.1093/haschl/qxaf056)

# Appendix

**Supplemental Figure 1.** Total Monthly Hospital Discharges Among Medicare Beneficiaries With High-Risk Comorbidities, 2018-2022. Total count of monthly hospital discharges among Medicare beneficiaries aged 65 and older with at least one of five selected comorbidities. First vertical dashed line indicates the onset of the COVID-19 pandemic in March 2020; second vertical dashed line indicates the introduction of COVID-19 vaccines in December 2020.


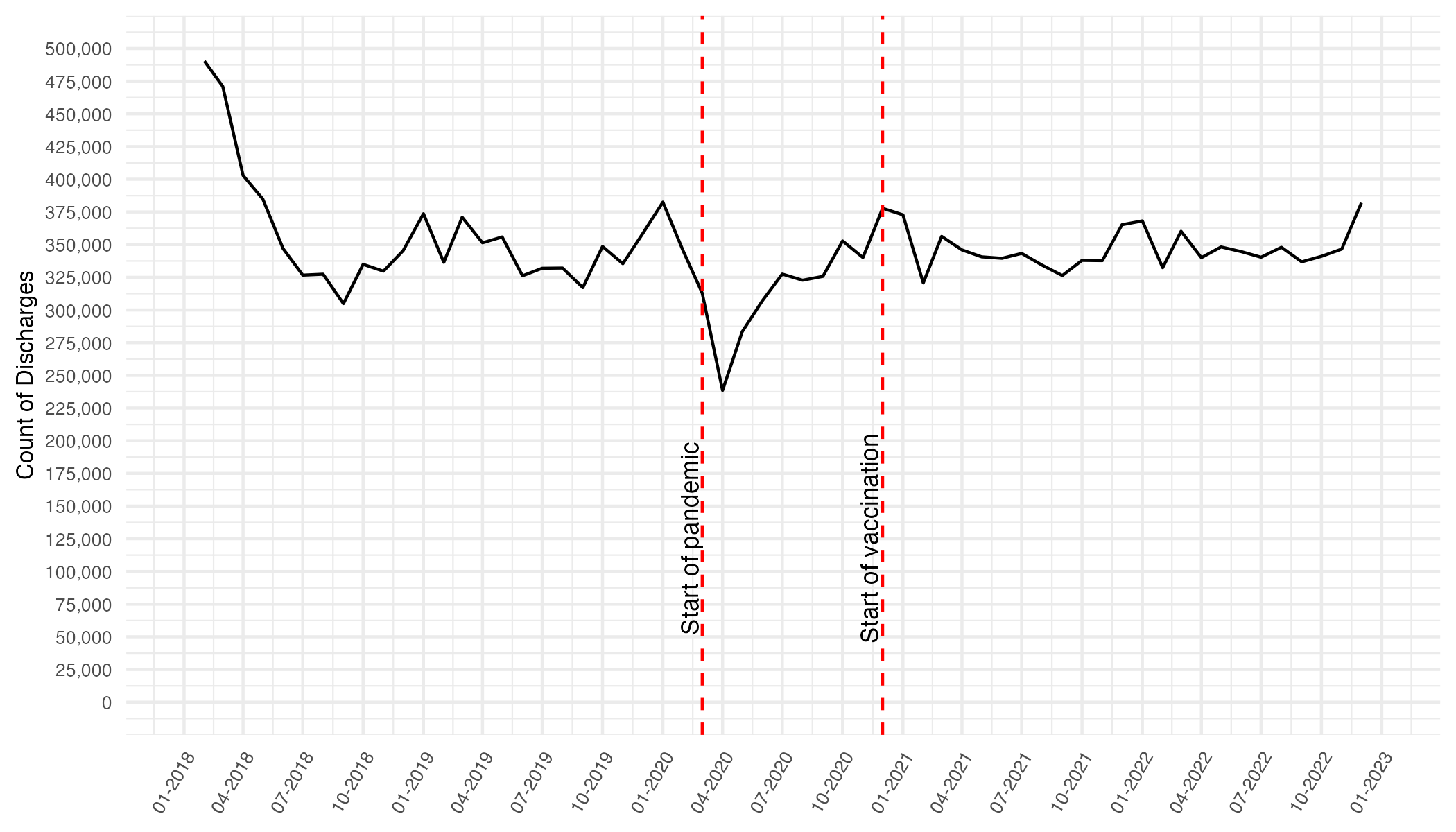


**Supplemental Figure 2.** Proportion of Hospital Discharges by Comorbidity Type Among Medicare Beneficiaries, 2018-2022. Monthly proportion of hospital discharges with each of the five selected comorbidities (dementia, diabetes, congestive heart failure, hip fracture, stroke) among Medicare beneficiaries aged 65 and older. First vertical dashed line indicates the onset of the COVID-19 pandemic in March 2020; second vertical dashed line indicates the introduction of COVID-19 vaccines in December 2020. Comorbidities identified using enhanced Chronic Conditions Data Warehouse ICD-10 code algorithms applied to all 25 diagnosis fields in Medicare Provider Analysis and Review data.


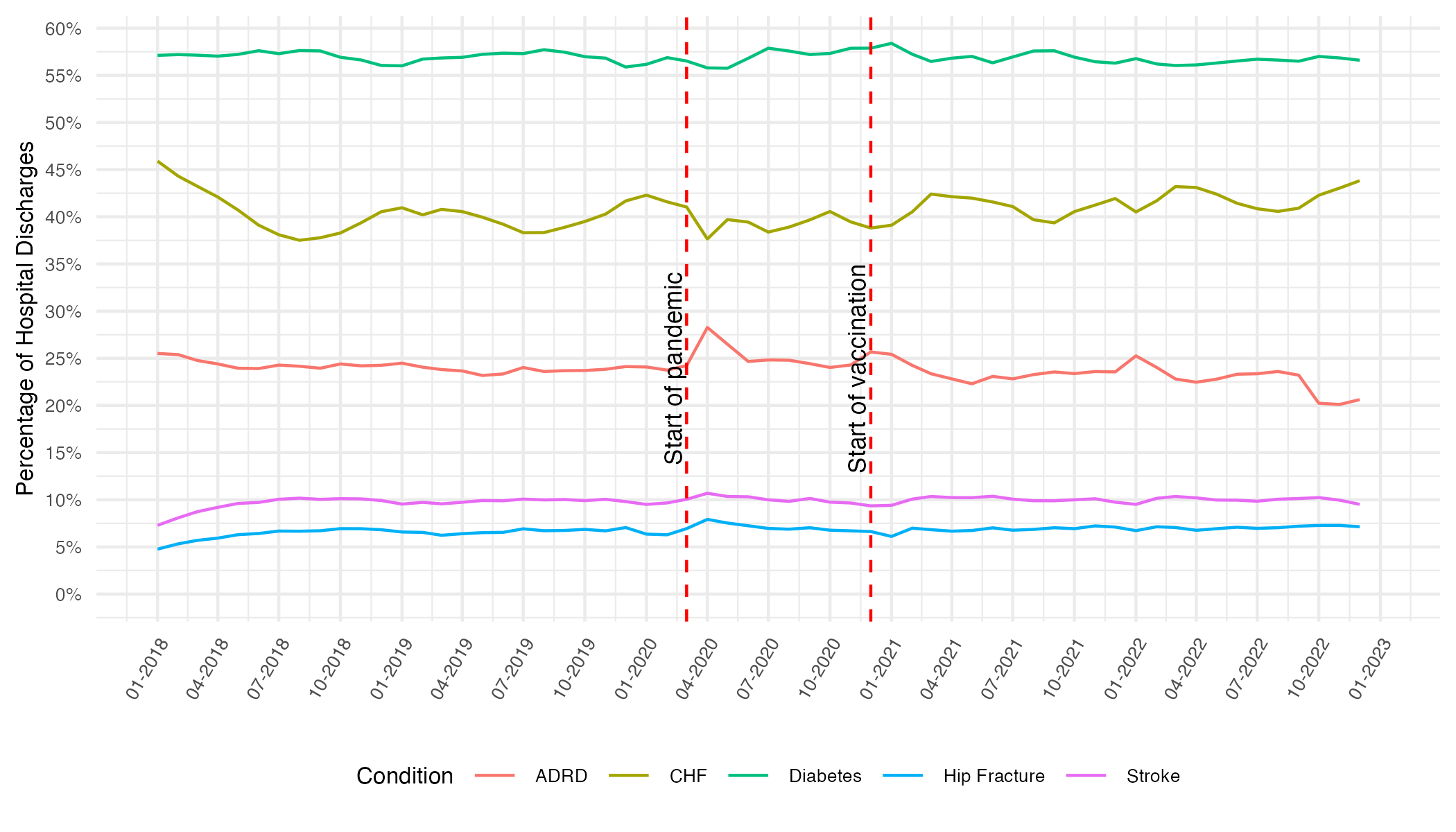


**Supplemental Figure 3.** Risk-Adjusted Probability of Discharge to Inpatient Rehabilitation Facilities Among Medicare Beneficiaries With High-Risk Comorbidities, by COVID-19 Status and Payer Type, 2018-2022. Monthly predicted probability of discharge to inpatient rehabilitation facilities from logit models with hospital fixed effects, adjusted for age, sex, race, dual eligibility status, Charlson Comorbidity Index, and comorbidity diagnoses. First vertical dashed line indicates the onset of the COVID-19 pandemic in March 2020; second vertical dashed line indicates the introduction of COVID-19 vaccines in December 2020. Shaded areas represent 95% confidence intervals.


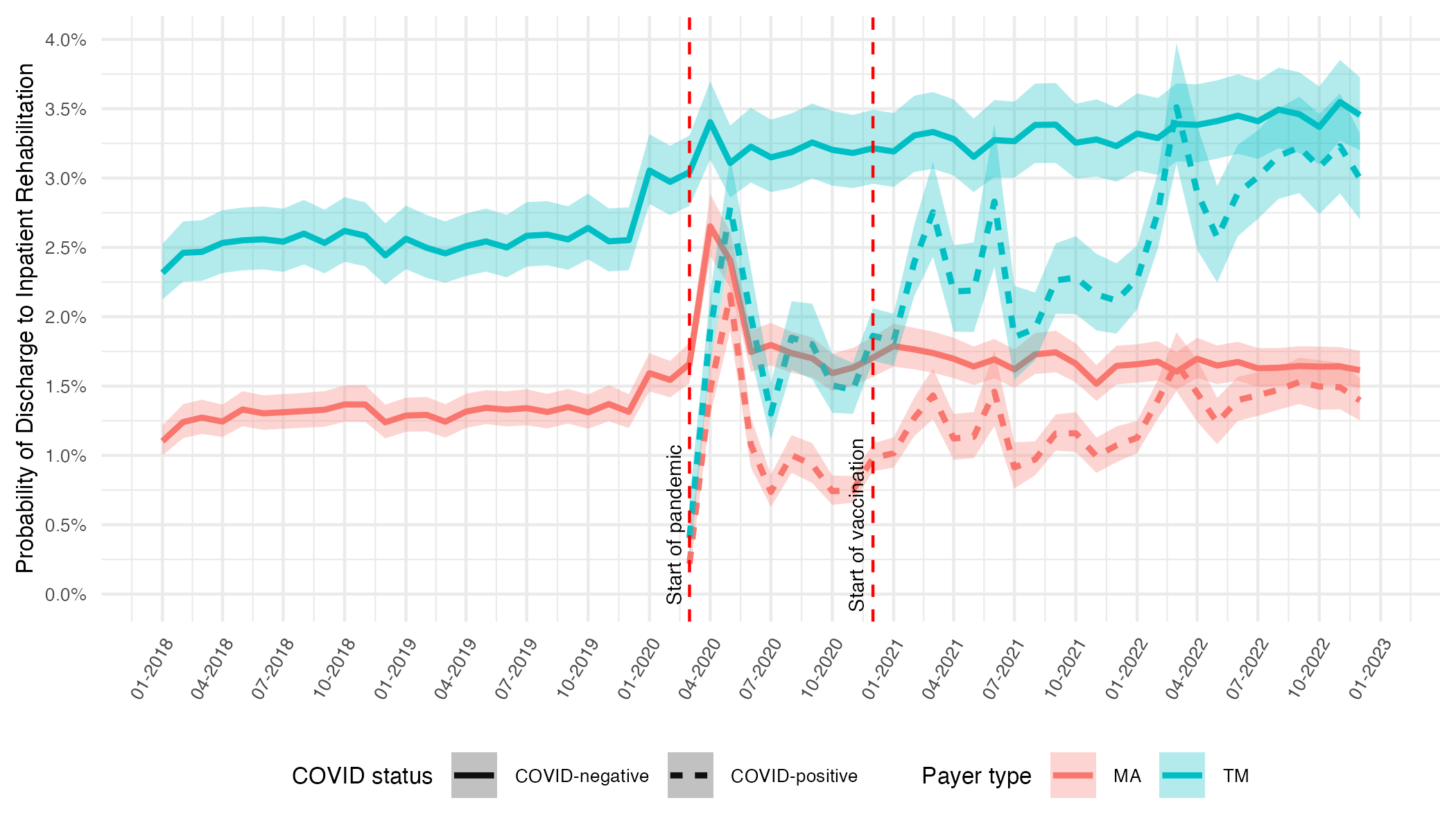

Supplement: qxaf056_Supplementary_Data [file qxaf056_supplementary_data.zip › Appendix_2025-03-06.docx]
